# The role of adipose tissue in puberty and reproductive health

**DOI:** 10.3389/fendo.2025.1543787

**Published:** 2025-06-17

**Authors:** Xin Ning, Qing Huang, Doudou Guo, Yanfen Zhou, Yating Li, Xin Li

**Affiliations:** ^1^ Department of Pediatrics, Union Hospital, Tongji Medical College, Huazhong University of Science and Technology, Wuhan, China; ^2^ International Medical College, Chongqing Medical University, Chongqing, China

**Keywords:** adipose tissue, puberty, adipocyte development, early life stage, reproductive function

## Abstract

Adipose tissue is an endocrine organ that signals energy status to the hypothalamic–pituitary–gonadal axis to regulate reproductive function. Notably, in mammals, adipose tissue biology—adipose tissue expansion and body fat distribution—is closely linked to the onset of puberty. Some studies showed that early adipose tissue development continues into childhood or adulthood, indicating its potential impact on reproductive function. Factors such as maternal obesity, childhood body mass index gain, and adolescent obesity significantly contribute to early puberty onset and negative reproductive events including menstrual irregularity, polycystic ovary syndrome, and male infertility. However, the connection between adipose tissue development before adulthood (prenatal stage and childhood) and reproductive function has not yet been fully investigated and reviewed. In this study, we present a comprehensive review of hormonal and inherent dimorphisms on adipose tissue development; there is a novel discussion about the link between adipose tissue expansion tracking throughout early life stages and reproductive disorders. Our study aims to elucidate how adipocyte development during critical periods of life can affect future reproductive health from sexual maturation to fertility and points to the clinical significance of further unlocking the underlying mechanism and weight management. As such, early prevention and long-term management for weight control might be considered as effective measures to mitigate obesity-induced reproductive comorbidities.

## 1 Introduction

Adipose tissue (AT) participates in a wide range of physiological and pathological processes such as energy metabolism, inflammation, reproduction function, and several types of cancers. Obesity, literally the excess of white adipose tissue (WAT), is a global concern of metabolic health that contributes to earlier pubertal development, impaired fertility, and polycystic ovary syndrome (PCOS) (1–3). With the rates of overweight and obesity increasingly growing in children, an increasing number of studies have revealed the prevalence of obesity-associated reproductive disorders in children and adolescents. Substantial studies have explored the connection among AT, puberty, and gonadal function. AT development begins in prenatal life and infancy, and tracks into childhood; it influences sexual maturation (4–7). Furthermore, childhood obesity is associated with precocious puberty, hyperandrogenism, suppression of gonadotropins, and the development of PCOS (8–11). This review discusses hormonal and inherent dimorphisms on AT development and provides a broad description of AT development during fundamental life stages and its role in puberty and later reproductive function.

## 2 The characteristics of adipose tissue

In mammals, AT primarily consists of WAT and brown AT (BAT). BAT plays a thermogenic role and is essential for maintaining body temperature. As an endocrine organ, it is considered as a metabolic pool for glucose, lipid, and branched-chain amino acids, and is related to metabolic diseases such as overweight and obesity (12). In humans, BAT mainly exists in fetuses and newborns to enhance neonatal survival, but decreases shortly after birth (13). WAT stores excess energy in the form of triglyceride and secretes various hormones that regulate energy metabolism. WAT is generally classified as subcutaneous AT (SAT) and visceral AT (VAT). SAT depots are mainly the abdominal, gluteal, and femoral types, but VAT, the so-called intraabdominal fat depot, is associated with internal organs. Women generally have more fat accumulation than men. Men accumulate more fat in the upper body (central obesity), which is associated with the development of cardiovascular disease, insulin resistance, and type 2 diabetes mellitus (14–16). Women accumulate more in the lower body (peripheral obesity), which protects against metabolic disorders (14).

AT development is a dynamic and proliferative process involving adipogenesis and remodeling through differentiated adipocyte precursor cells (APCs). The expansion of fat mass can occur through an increase in the average size of fat cells and/or number of adipocytes. In adults, enlarged fat cells are a key feature of increased fat storage in fat depots, whereas in children with obesity, there is a significant increase in both adipocyte number and cell size (17–19). From 6 months to 1 year of age, cell size increased to adult levels and then decreased until 2 years of age, during which larger adipocytes contributed to an increase in fat depots. After 2 years of age, cell size did not change significantly until early adolescence age (19). Adipocyte number significantly increases only after age 10. In contrast, significant increases in cell number and size in obese children were observed throughout all ages (19). Overall, the changes in adipocytes for childhood obesity are characterized by adipocyte hypertrophy and hyperplasia.

## 3 The hormonal and inherent dimorphisms on adipose tissue development during puberty

Puberty is another important period for the hypercellularity of AT. For mammalian females, body fat increases during puberty onset and is predominantly localized in gluteofemoral fat depots, which is profoundly associated with the start of menarche (20, 21). For men, they also gain body fat during puberty, but lean mass mostly increases before a growth spurt to deplete body fat (22). This makes AT less remarkable on male pubertal development; however, it is still important in male puberty initiation (23). Sex differences exist in body fat distribution during puberty. In men, an android shape, which is mainly fat in the abdominal area, develops principally during this period. In women, fat is centered on the hips and remains gynecoid during puberty.

Sex steroid hormones (estrogen and androgen) are considered to be the main cause of sex differences in fat distribution. The mechanism underlying the sex- and depot-specific fat distribution remains poorly understood. Gonadal hormones, including estrogen (E2), progesterone, and androgen, have their receptors expressed in both VAT and SAT depots. In women, SAT has higher expressions of estrogen receptors (ERs) and progesterone receptors (PRs) than androgen receptors (ARs). Estrogen promotes subcutaneous fat depot only after sexual maturation in women (24). Moreover, its receptor ERα signaling in women leads to subcutaneous fat accrual and the reduction of visceral adipocyte mass, but ERβ may inhibit the effect of E2 on adipocytes after sex maturation (25–27). In both female and male mouse models, epididymal, perirenal, and inguinal WAT weighed more in ERα knockout (αERKO) mice than in their wild-type control (28). Moreover, androgen and adrenal steroids such as dehydroepiandrosterone (DHEA), dehydroepiandrosterone sulfate (DHEAS), and some subtypes of 17-β hydroxysteroid dehydrogenase (HSD) isoenzymes are also associated with body fat distribution in women. Visceral adiposities were positively associated with omental 20α-HSD level and 3α-HSD-3 level (29). However, the association between plasma androgen levels and visceral fat accumulation is not always consistent.

As in female AT, ER is also expressed in male AT. Estrogen treatment decreased adipocyte size in male rats (30). αERKO male mice showed increased WAT, especially in epididymal, perirenal, and inguinal depots (28). In men, visceral fat accumulation is inversely associated with circulating testosterone (cT) levels and sex hormone-binding globulin (SHGB) (31–33), and testosterone treatment decreases abdominal subcutaneous and gluteal depots in female-to-male transsexuals (34). Additionally, androgen receptor knockout (ARKO) mice develop late-onset visceral adiposity and total fat mass (35–37).

Several lines of evidence suggested that local androgen metabolism in AT could affect body fat distribution. DHEA, an adrenal precursor to the formation of active steroids, has been found to be negatively associated with abdominal fat accumulation in men (38). Peripheral androgen metabolites (PAMs), such as 5α-androstane-3α and 17β-diol glucuronide (3α-diol-G), are positively associated with visceral fat accumulation (39). Steroid-inactivating enzymes, such as the aldoketoreductase 1C (AKR1C) family, which are mostly responsible for androstenedione (DHT) inactivation, are highly expressed in AT and are positively related to omental adipocyte size and visceral fat accumulation (29, 40–42). From the evidence above, local androgens might play a more significant role in fat accumulation than circulating androgens in men.

**Figure 1:**
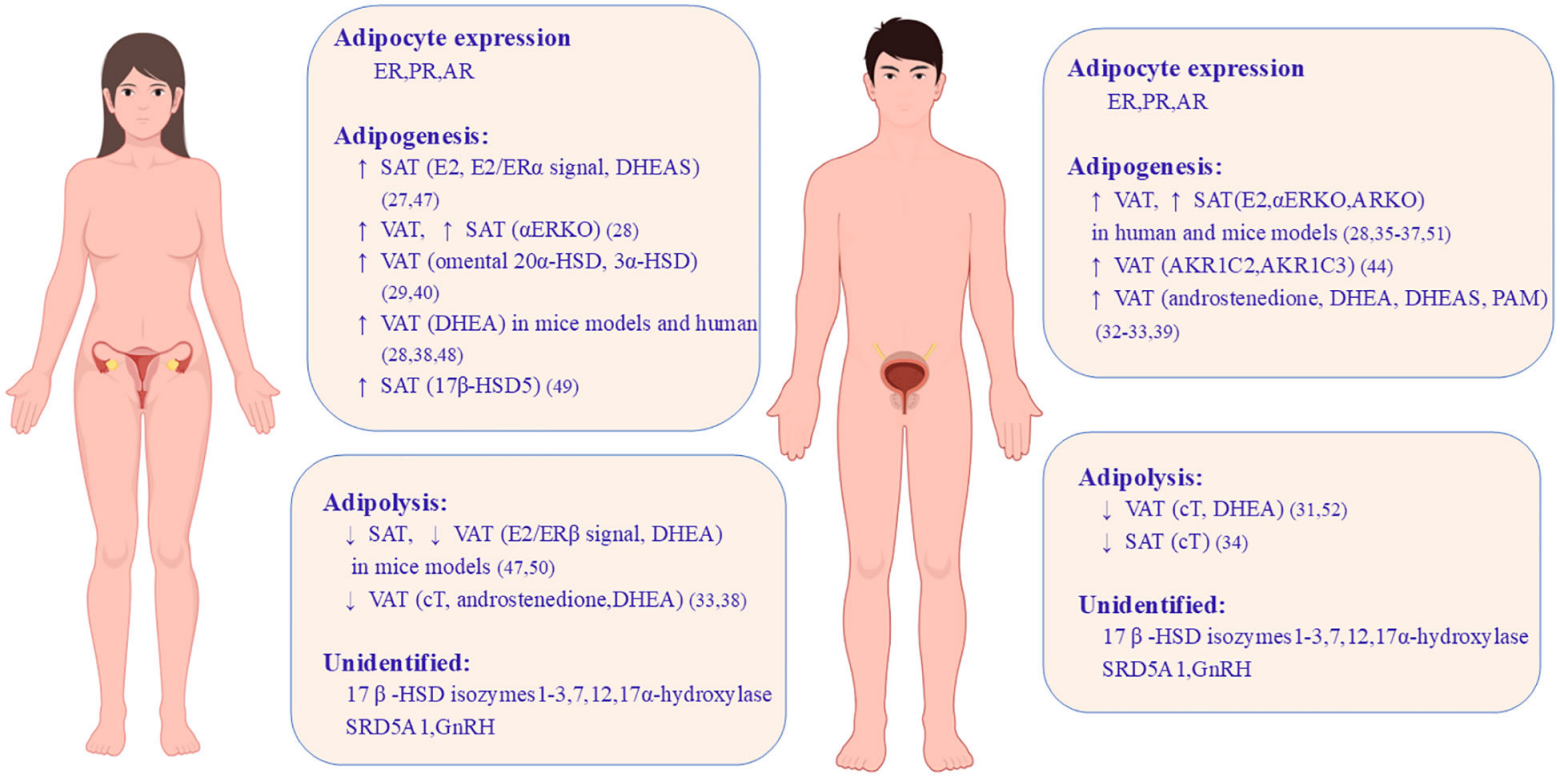
Sex differences caused by gonadal hormones and steroid-metabolizing enzymes on adipose tissue development. Both women and men have ER, PR, and AR expressed in adipocytes but their role in adipogenesis and adipolysis shows sex differences and depot differences. This image shows the roles of sex hormones, their cognate receptors, and steroid-metabolizing enzymes in VAT and SAT in mice models and human. ↑, increase; ↓, decrease; ER, estrogen receptor; E2, estrogen; PR, progesterone receptor; AR, androgen receptor; VAT, visceral adipose tissue; SAT, subcutaneous adipose tissue; DHEA, dehydroepiandrosterone; DHEAS, dehydroepiandrosterone sulfate; ERKO, estrogen receptor knockout; ARKO, androgen receptor knockout; HSD, hydroxysteroid dehydrogenase; cT, circulating testosterone; SRD5A1, 5α-reductase type 1; AKR1C2/AKR1C3, aldoketoreductase 1C family; PAM, peripheral androgen metabolites (47–52).

Some androgen-metabolizing enzymes are identified in AT and might indirectly contribute to depot differences. 5α-Reductase type 1 (SRD5A1), an androgen-metabolizing enzyme, and its metabolites increased with obesity in humans (43–45). Other steroid-metabolizing enzymes, such as 3β-hydroxysteroid dehydrogenase (HSD) type 1; 17β-HSD types 2, 3, 7 and 12; and 17α-hydroxylase, have also been detected in AT, but their role in fat depots remains unknown. Furthermore, evidence has shown an association between gonadotropin levels and adipocytes. Li et al. discovered that gonadotropin-releasing hormone receptors (GnRHRs) are expressed in human adipocytes, and the activation of GnRHR could increase the cell number of preadipocytes and the accumulation of lipid droplets by inhibiting AMPK pathways (46). Thus, gonadal hormones, steroid hormones, and their metabolizing enzymes are likely to participate in adipocyte differentiation. Regional variation and intra-adipocyte hormone metabolism might explain the heterogeneity of hormonal effects on fat distribution. The roles of sex hormones, their receptors, and steroid-metabolizing enzymes in adipogenesis and adipolysis within fat depots are concluded in **Figure 1**.

Except for gonadal hormones, sex chromosomes might inherently determine sex differences in adipocyte biology. Gonadectomized mice with XX versus XY gain more weight and adiposity, particularly inguinal WAT, indicating the effect of X chromosome on weight gain independent of gonadal steroids (53). These genetic studies show that an increased number of X chromosomes, rather than the Y chromosome, leads to differences in adiposity. The presence of two X chromosomes (XX and XXY mice with gonadectomy) led to higher body weight/fat than one X chromosome (XY and XO mice with gonadectomy), while the presence of the Y chromosome did not have an effect (53). Thus, these results implicated the X chromosome gene as a direct cause of sex differences in fat distribution.

Sexually dimorphic genes and epigenetic modification in gene expressions have been identified as contributing factors in AT biology. A review of these genes can be found in references (54–56). In conclusion, sex differences in body fat distribution during puberty are not solely determined by the secretion of gonadal hormones, but rather by a more complex interplay of depot-specific adipocyte differentiation, hormonal signaling, and genetic modifications.

## 4 The association between adipose tissue during early life stage and puberty

The development and expansion of AT begins in the fetus and extends throughout the lifespan. Rapid fat accrual occurs during the late prenatal period and infancy. Studies have indicated that adiposity in prenatal life and infancy tracks into childhood, and is associated with childhood obesity and body composition (4, 5, 57–59). Thus, the link between adiposity during this period and puberty has drawn much attention. Clinical studies have shown that early weight gain in infancy and the trajectories of body mass index (BMI) percentage during early childhood predict younger ages at menarche and the onset of breast development (7, 60–63). In a retrospective longitudinal study, the timing of menarche and thelarche showed an inverse relationship with the change in *z*-score from birth weight (64). Furthermore, restricted fetal growth and prenatal maternal fat also affect pubertal development. Girls born small-for-gestational age (SGA) reach all pubertal markers at an earlier mean age than those born appropriate-for-gestational age (AGA), except for breast development, while boys born SGA and large-for-gestational age (LGA) achieved puberty earlier than those born AGA (6).

**Figure 2:**
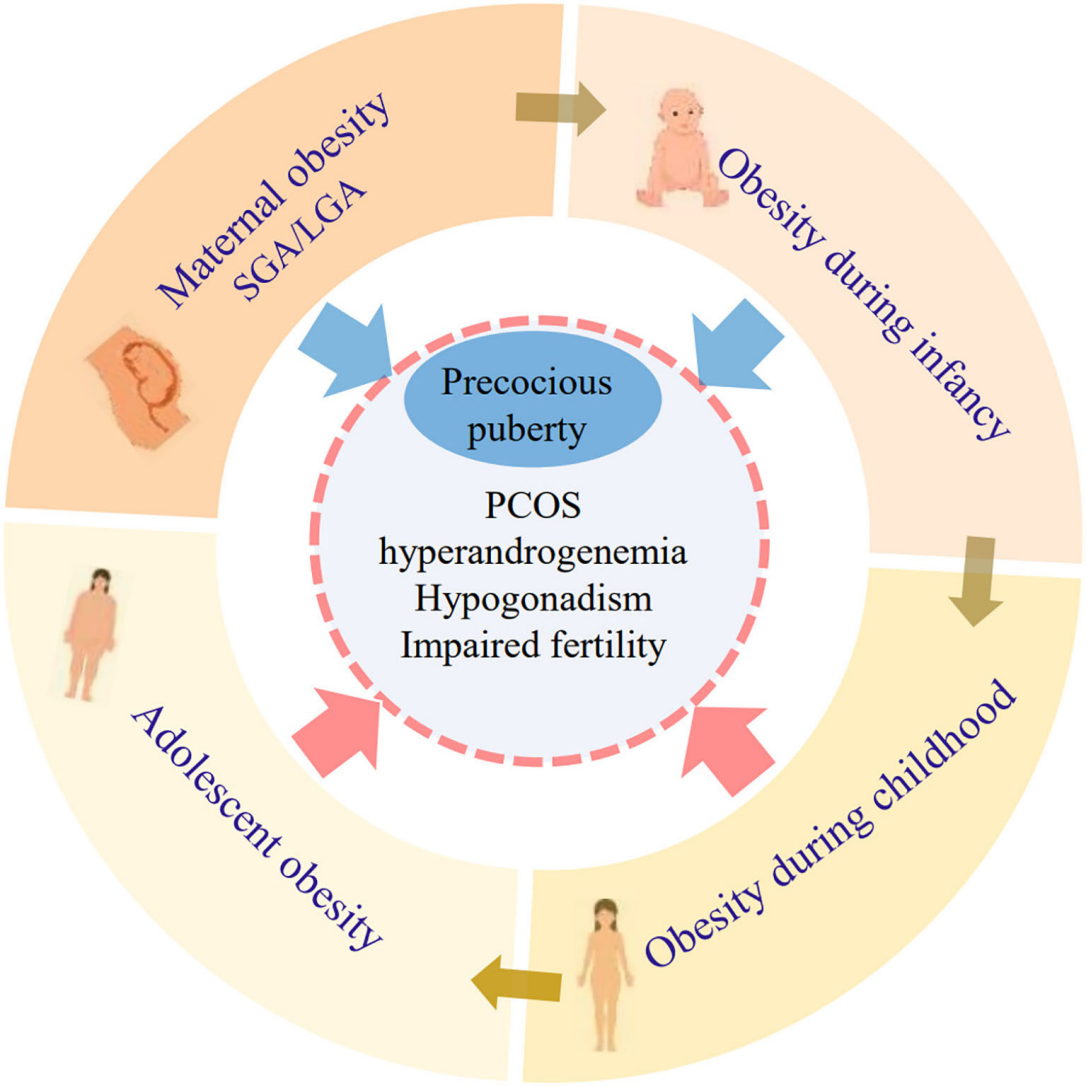
The connection between obesity during critical life stages and reproductive disorders. Adiposity at each developmental stage prior to puberty onset might last toward the next life stages. Perinatal factors (maternal obesity, SGA, LGA, and infant obesity) are closely related to precocious puberty. Obesity during childhood and adolescence is related to high risks of multiple reproductive disorders such as PCOS, hyperandrogenemia, hypogonadism, and impaired fertility. SGA, small for gestational age; LGA, large for gestational age; PCOS, polycystic ovary syndrome.

Maternal obesity, even before pregnancy, is associated with earlier pubertal development in offspring (65–72). The mechanism linking maternal fat to offspring puberty remains to be elucidated. In rat models, maternal high-fat diet during the early postnatal period induced increased Kiss1 expression in the ARC and early puberty onset in female offspring (73). A study conducted by Lam et al. found that MC3R, expressed in KNDy neurons of the hypothalamic arcuate nucleus, could be activated as an intermediary signaling pathway to relay nutritional status to childhood growth and the timing of puberty (74). Increased endogenous estradiol in the progeny of obese rats is associated with precocious puberty and altered follicular development in adulthood (75). These findings underscore the importance of AT development during the prenatal period and childhood in determining their susceptibility to early pubertal maturation (**Figure 2**).

## 5 The association of adipose tissue during childhood and adolescence and puberty

Puberty is a critical process of sexual maturation and is characterized by a growth spurt. Prior to puberty, there are minimal sex differences in body composition, but differences in AT development become more apparent from puberty. Sexual dimorphism in regional fat patterning emerges, with girls exhibiting less waist and more hip fat than boys from puberty to early adulthood (76–78). Several studies have established a link between childhood adiposity and puberty. Childhood obesity is closely associated with earlier sexual maturation in girls (79, 80), whereas the relationship between obesity and pubertal timing in boys remains controversial (79, 81). A growing number of studies have shown that obesity in boys is also associated with early puberty onset (80, 82–86). The onset and progression of puberty in boys are positively related to weight and BMI (87). In a body composition analysis study, boys with high-level percentage of body fat (BFP) had an increased risk of earlier pubertal onset (88). These results might be attributable to the accurate assessment of testicular volume using a Prader orchidometer and BMI *z*-score or body composition metrics as indicators of body fat. Despite the link between adiposity during childhood and pubertal development, there is a lack of data on the link between body composition in children aged 2 to 5 years and the onset of puberty. This gap is largely due to the lack of age-specific body composition evaluation and a longer follow-up period.

## 6 Adipose tissue before puberty and adulthood reproductive outcomes

Overweight or obesity during childhood and adolescence is associated with impaired reproductive functions. In girls, obesity, especially central obesity, is associated with a high risk of menstrual irregularity and PCOS (10, 89–92). Weight loss is associated with improvement in PCOS symptoms. Among prepubertal girls, BMI is significantly and positively associated with free testosterone, and obese girls have a high risk of hyperandrogenemia. A cohort study recruiting individuals followed from birth to age 50 years (93) indicated that, in early childhood (age 3–6 years), there was no significant association between underweight, overweight, or obesity and any fertility outcomes, but obesity at age 11–15 years was associated with a higher risk of decreased fecundability and childlessness in adulthood, independent of PCOS.

Unlike girls, there is less evidence about the direct correlation between AT before puberty and male reproductive dysfunction. In male infertility, obesity is associated with disrupted spermatogenesis, reduced semen quality, and erectile dysfunction (94, 95). Several studies have reported a link between hypogonadism and obesity in adolescent men (96, 97). However, it remains unclear whether reproductive impairments related to obesity begin during childhood or adolescence. Genetic obesity syndromes offer new insight that early-onset obesity is presumably a contributing factor for reproductive impairments as obesity syndromes (e.g., Prader–Willi syndrome, Cohen syndrome, and Bardet–Biedl syndrome) often present with intractable obesity at an early age as well as hypogonadism and irregular menses (9, 98). The relationship between obesity during critical life stages and reproductive disorders is detailed in **Figure 2**. Conversely, Ramlau-Hansen et al. found that prepubertal BMI was not significantly associated with semen quality (99). The inconsistency might result from the lack of longitudinal evaluation of BMI change, not excluding the possibility of weight loss before semen collection. Little evidence from rat models has explored the effects of prepubertal obesity on gonadal function. In a rat model, prepubertal obesity resulted in a reduced number of Leydig cells, the decreased expressions of steroidogenic acute regulatory protein (StAR), and compromised ovarian oxidative stress and DNA repair (100, 101). These preliminary data provide a possibility that inconspicuous lesions exist in reproductive systems in the context of exposure to early-onset overweight or obesity.

## 7 Discussion

AT distribution is shaped by gonadal hormones, steroid-metabolizing enzymes, and genetic modification during puberty; AT development during early life stages has a close relationship with puberty timing and later reproductive function. Substantial evidence has shown that obesity (maternal obesity or obesity during infancy, childhood, and adolescence) contributes to high risks of precocious puberty, PCOS, and impaired fertility. Thus, weight management during early life stages should receive more attention, as it might be effective to improve reproductive outcomes in young adults. However, the mechanisms underlying sexual dimorphism in fat distribution, particularly the role of gonadal and steroid hormones in mediating depot-specific variations across distinct adipose depots, remain to be fully elucidated. In clinical practice, the early identification of obesity-associated reproductive dysfunction remains unclear, and there is still lack of evidence to assess the long-term impact of childhood obesity—whether transient or persistent—on adult reproductive outcomes. Future studies should focus on clarifying the mechanism whereby adiposity during early life stages leads to impaired reproductive function. Longitudinal cohort studies are needed to assess the effectiveness of weight management interventions in improving reproductive outcomes during young adulthood.
